# The CSF lipid profile in patients with probable idiopathic normal pressure hydrocephalus differs from control but does not differ between shunt responders and non-responders

**DOI:** 10.1093/braincomms/fcae388

**Published:** 2024-11-05

**Authors:** Trine L Toft-Bertelsen, Søren Norge Andreassen, Anja Hviid Simonsen, Steen Gregers Hasselbalch, Nanna MacAulay

**Affiliations:** Department of Neuroscience, University of Copenhagen, 2200 Copenhagen, Denmark; Department of Neuroscience, University of Copenhagen, 2200 Copenhagen, Denmark; Department of Neurology, Section 6911, Danish Dementia Research Centre, Copenhagen University Hospital - Rigshospitalet, 2100 Copenhagen, Denmark; Department of Neurology, Section 6911, Danish Dementia Research Centre, Copenhagen University Hospital - Rigshospitalet, 2100 Copenhagen, Denmark; Department of Clinical Medicine, Faculty of Health and Medical Sciences, University of Copenhagen, 2200 Copenhagen, Denmark; Department of Neuroscience, University of Copenhagen, 2200 Copenhagen, Denmark

**Keywords:** CSF, iNPH, lipidomics, mass spectrometry

## Abstract

Idiopathic normal pressure hydrocephalus is a common form of hydrocephalus in the elderly, characterized by enlarged ventricles combined with clinical symptoms presenting as gait impairment, urinary incontinence, and dementia. Idiopathic normal pressure hydrocephalus may be difficult to differentiate clinically from other neurodegenerative disorders, and up to 80% of cases may remain unrecognized and thus untreated. Consequently, there is a pressing demand for biomarkers that can confirm the diagnosis of idiopathic normal pressure hydrocephalus. In this exploratory study, CSF was sampled from the lumbar compartment of 21 control individuals and 19 probable idiopathic normal pressure hydrocephalus patients and analyzed by an untargeted mass spectroscopy-based platform to reveal a complete CSF lipid profile in these samples. Two hundred forty-four lipids from 17 lipid classes were detected in CSF. Various lipid classes, and select individual lipids, were reduced in the CSF obtained from patients with probable idiopathic normal pressure hydrocephalus, whereas a range of lipids belonging to the class of triacylglycerols was elevated. We detected no difference in the CSF lipid profile between probable idiopathic normal pressure hydrocephalus patients with and without clinical improvement following CSF shunting. In conclusion, the lipidomic profile of the CSF in patients with probable idiopathic normal pressure hydrocephalus, therefore, may serve as a sought after biomarker of the pathology, which may be employed to complement the clinical diagnosis.

## Teaser

To determine whether CSF lipids can be used as biomarkers of idiopathic normal pressure hydrocephalus, Toft-Bertelsen *et al*. utilized liquid chromatography/tandem mass spectrometry to assess the lipid composition in both patients and controls. Toft-Bertelsen *et al*. report that the lipidomic signature may be employed to complement the clinical diagnosis.

## Graphical Abstract

**Graphical Abstract:**
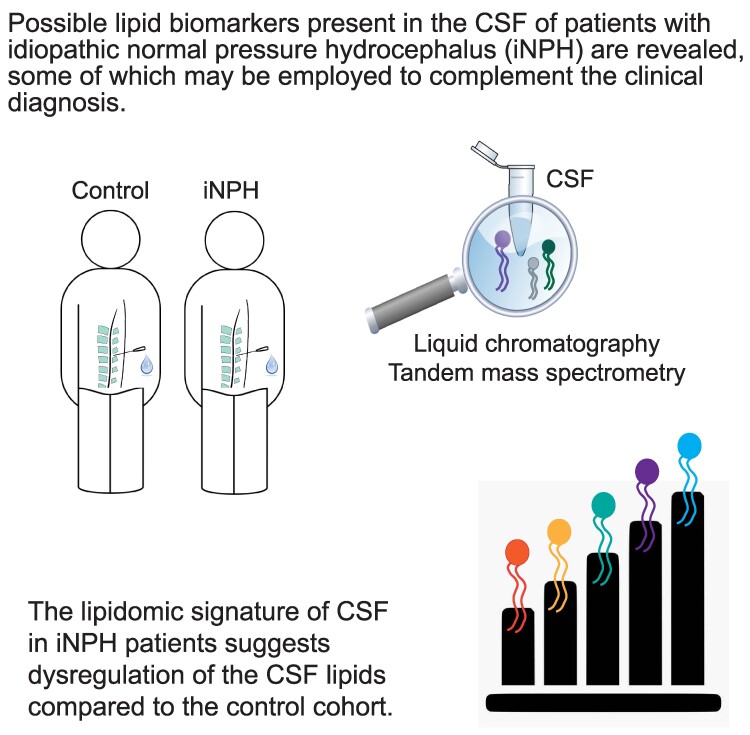

## Introduction

Idiopathic normal pressure hydrocephalus (iNPH) is a neurological condition that predominantly affects individuals aged 65 years and older^1^ and is characterized by a triad of clinical symptoms, including cognitive decline, gait disturbances, and urinary incontinence.^2^ Patients with iNPH, in addition, present with ventricular enlargement, a central element in the clinical syndrome.^3^ Nevertheless, iNPH has proven challenging to distinguish from other neurodegenerative diseases, such as Alzheimer’s disease.

Shunt insertion to drain excess CSF often results in an improvement in the patient's clinical condition,^4^ which suggests that CSF accumulation is a component of the condition's aetiology. Unfortunately, surgical shunting comes with inherent risks, such as infection and shunt failure, and should therefore only be employed with predicted benefit for the patient. With the difficulty in precise diagnosis of iNPH patients and subsequent prediction of shunt responsiveness, it would be beneficial to obtain biomarkers that could cement the diagnosis of iNPH, predict which patients respond favourably to CSF diversion and those who do not, and/or reveal the biological mechanisms of injury and recovery in iNPH.

CSF has garnered significant interest as a biomarker matrix for diagnosing and treating iNPH, and this patient group may indeed exhibit unique CSF composition changes compared with control individuals, as illustrated with quantification of inflammatory markers and proteomics,^5-7^ although with no promising biomarker revealed so far. As a complementary alternative, lipidomics offers a comprehensive analysis of lipids within a specific compartment and is well suited for the determination of CSF lipid imbalance in brain-related disorders. Emerging evidence suggests an important role for lipids in many neurodegenerative processes,^8-11^ although with only one report of a full CSF lipidomic profiling of patients with subarachnoid haemorrhage,^12^ and the remaining few studies on CSF lipid changes performed on select lipids, i.e. sphingolipids, ceramides, sphingomyelins (SMs) and glycolipids.^5,6,13^ We therefore here utilized liquid chromatography coupled with tandem mass spectrometry, which is a highly effective analytical platform for untargeted lipidomics offering exceptional precision and resolution. We applied this technique to assess the lipid composition of CSF collected from both probable iNPH patients and control individuals and to determine whether the delineated lipid pool could be used as a biomarker of iNPH and to predict the shunt outcome.

## Materials and methods

### Patients

CSF samples were collected at the Danish Dementia Research Centre, Copenhagen University Hospital – Rigshospitalet, Denmark between May 2011 and July 2018 from 19 patients (range 64–84 years, 7F/12M) diagnosed with probable iNPH according to the international guidelines from 2005.^3^ The patients were evaluated for cognitive impairment (scored by mini-mental state examination),^14^ gait/balance disturbances and urinary incontinence (validated clinical scales were used for scoring),^15^ brain imaging, and CSF opening pressure. CSF samples were collected before the infusion test during diagnostic examination [infusion test with subsequent tap test using the CELDA system (Likvor, Umea, Sweden) through two lumbar needles]. All enrolled patients with probable iNPH had either an abnormal infusion test or a positive tap test to support the diagnosis and therefore received a ventriculo-peritoneal shunt. Eleven probable iNPH patients were designated as ‘responders’ due to improvement in clinical symptoms following ventriculo-peritoneal shunt insertion. The remaining eight probable iNPH patients were categorized as ‘non-responders’, having shown no significant clinical improvement post-surgery. Shunt response was reassessed 6 months after surgery using the same clinical scales.^16^ The inclusion criteria for the iNPH group were (i) sample availability from the biobank, (ii) shunt surgery performed at the Department of Neurosurgery, Rigshospitalet and (iii) documented follow-up data on shunt response. Twenty-one individuals (range 52–84 years, 8F/13M) were referred on suspicion of cognitive dysfunction or referred for evaluation. These individuals were evaluated with normal cognition and no neuroimaging suggesting brain disorder (incl. iNPH) and were thus enrolled as healthy individuals serving as control subjects. CSF samples were centrifuged at 2000*g* for 10 min at 4°C within 2 h from collection and stored at −80°C in the biobank. Written informed consent was obtained from all patients and control subjects or next of kin depending on the capacity of the individuals. The study was approved by the ethics committee of the Capital Region of Denmark (H-18046630) and the Danish Data Protection Agency (VD-2019-210).

### Liquid chromatography with mass spectrometry analysis

Liquid chromatography with mass spectrometry was performed according to Toft-Bertelsen *et al*.^12^ Briefly, CSF was processed using a Spin-X® filter and centrifuged before transferring into a high-performance liquid chromatography vial for analysis by liquid chromatography with mass spectrometry. Lipid separation was done using a Waters® ACQUITY C18 column, with a two-step gradient. MS analysis was performed in both ionization modes. Data processing involved Compound Discoverer 3.0, with quality control using internal lipid standards. Three levels of annotation were applied, excluding Level 3 in this study.

### Bioinformatics and statistical analysis

Bioinformatics and statistical analysis were performed according to Toft-Bertelsen *et al*.^12^ Briefly, a Smirnov–Grubbs test (two-sided, *α* = 0.05) was applied to 358 compounds in control (*n* = 21) and probable iNPH (*n* = 19) groups. Samples with >20% outliers were excluded (control = 1, iNPH = 1). Non-biological lipid compounds and non-classified (‘others’) were excluded from the analysis. After curation, 244 compounds across 17 lipid groups were analyzed. Lipid abundance was normalized to quality controls (*n* = 15), and enrichment plots were generated. Principal component analysis (PCA)^17^ was used for cohort separation based on sex, age, iNPH status and lipid group. Statistical analysis included Welch's *t*-test with Benjamini–Hochberg correction (false discovery rate = 5%).^18,19^ Full analysis scripts are available at https://github.com/Sorennorge/MacAulayLab-iNPH-Metabolomics.

## Results

### CSF lipid composition

To characterize the lipid composition in lumbar CSF from patients diagnosed with probable iNPH, we utilized lumbar CSF samples obtained from 19 patients during their diagnostic examinations in comparison with lumbar CSF samples from patients referred on suspicion of cognitive dysfunction but were evaluated with normal cognition and no neuroimaging suggesting brain disorder (see Materials and methods for details). The latter samples are referred to as ‘control’ from here on. We utilized a non-targeted liquid chromatography with mass spectrometry approach to determine the CSF lipid composition in both groups. In total, we detected 358 different compounds, of which 244 were categorized into 17 distinct groups based on their primary lipid class (Supplementary Table 1). Groups containing fewer than four lipids (accounting for <1% of the total lipids identified) were consolidated into a single category referred to as the ‘small group collection’ (Supplementary Table 1). For the purposes of our study, molecules that could not be classified and non-biological lipids, such as various sugars, amino acids, vitamins and medications used in clinical practice (Supplementary Table 1), were excluded from further analysis. We observed no sex-dependent distribution of CSF lipids in control individuals or probable iNPH patients (Supplementary Fig. 1), but an apparent biphasic age-dependent lipid profile of most lipid classes, with the exception of the classes of fatty acids and monoacylglycerols that appeared relatively stable across age (Supplementary Fig. 1).

### Altered CSF lipid profile in patients with probable iNPH

**Figure 1:**
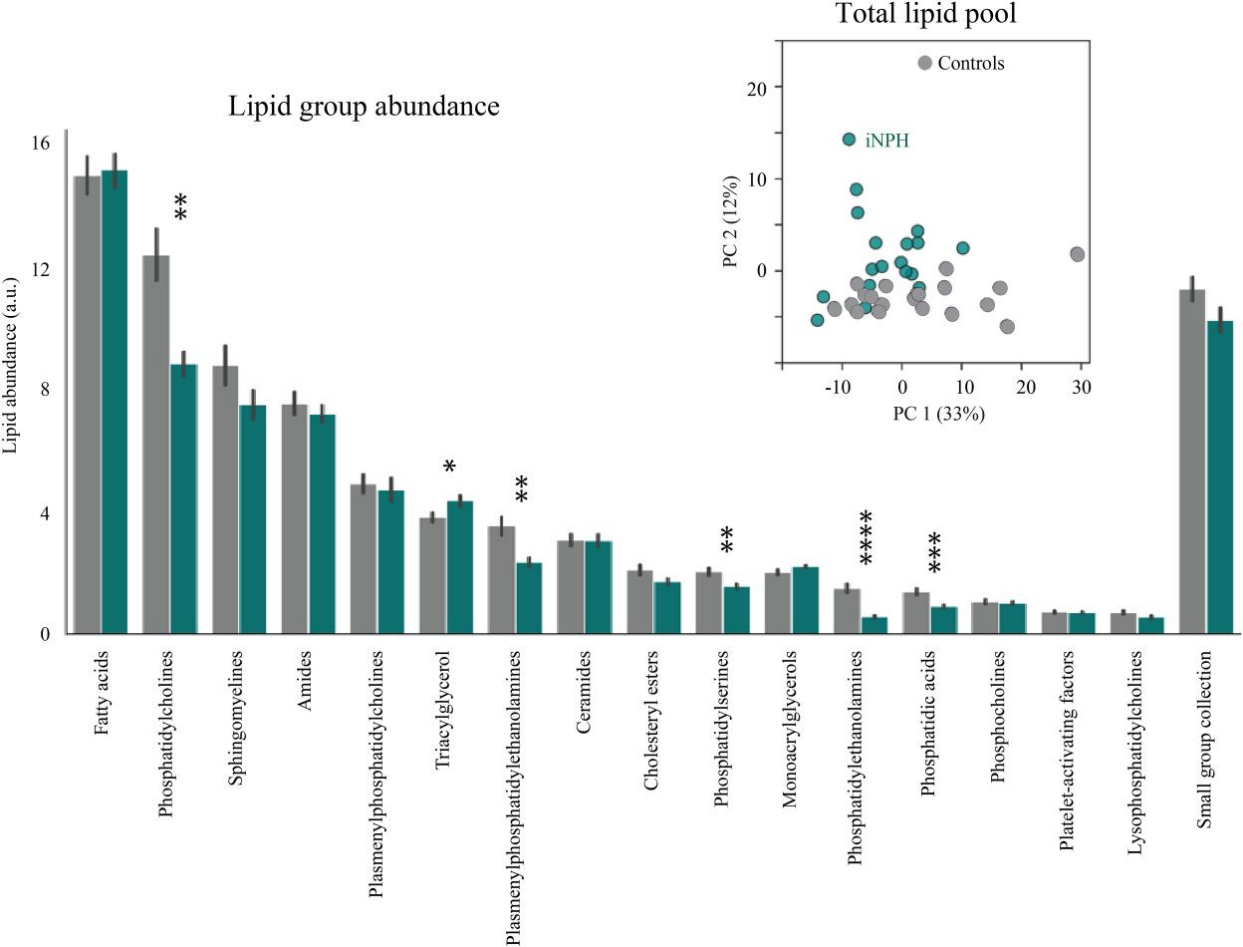
**Altered CSF lipid class profile in patients with probable iNPH.** The concentration of each lipid class in CSF from control subjects and patients with probable iNPH was plotted as bars (arranged according to their descending abundance) with the total lipid values for control subjects and patients with probable iNPH arranged in a PCA plot (inset).

**Figure 2:**
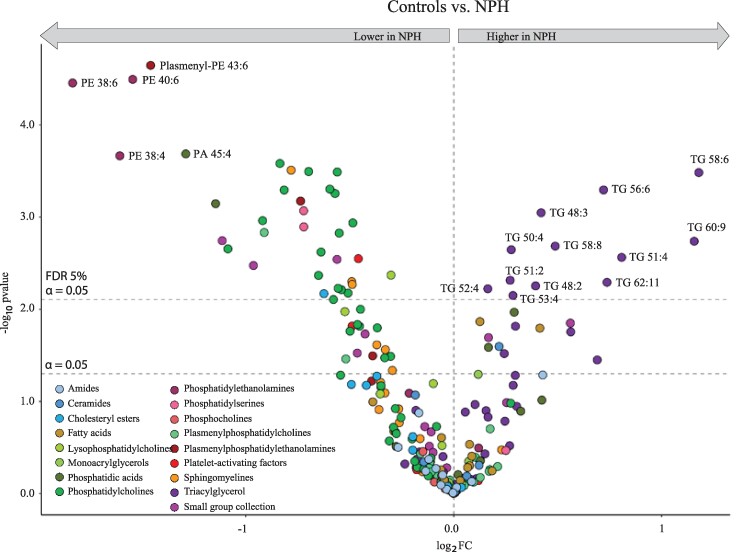
**Altered CSF lipid profile in patients with probable iNPH.** Volcano plot of individual CSF lipids identified with the fold change (log_2_ transformed) between control subjects and patients with iNPH. The upper dashed line indicates cut-off for significance (at a false discovery rate of 5%, ≤0.05). Statistical evaluation was done with Welch’s *t*-test followed by the Benjamini–Hochberg method (with an adjusted *P* ≤ 0.05). *P < 0.05, **P < 0.01, ***P < 0.001 and ****P < 0.0001. Data are based on lumbar CSF from 19 patients with probable iNPH and lumbar CSF from 21 control subjects. FC, fold change.

We observed a tendency, but not statistically significant, towards a lower lipid concentration in patients with probable iNPH (4.3 ± 0.2 a.u. in patients with probable iNPH versus 4.8 ± 0.2 a.u. in control subjects, *P* = 0.05). A PCA approach indicated a tendency towards a separation of the control CSF lipid content from that of the probable iNPH patients, indicative of non-identical CSF lipid profiles within the two groups (Fig. 1, inset). To identify individual lipid classes that may drive a diversion of the overall probable iNPH lipid profile from the control subjects, we compared the concentration of different lipid classes between the probable iNPH patients and control individuals (Fig. 1). Phosphatidylcholines (PCs), phosphatidylserines (PSs), phosphatidylethanolamines (PEs), plasmenylphosphatidylethanolamines (plasmenyl-PEs) and phosphatidic acids (PAs) were significantly lower and TGs higher in the CSF from patients with probable iNPH. To reveal alterations in individual lipid distribution in patients with probable iNPH compared with control individuals, the data were subsequently arranged in a volcano plot. Twelve lipids were significantly higher in the CSF from patients with probable iNPH, after correction for multiple testing (Fig. 2), all belonging to the lipid class of triacylglycerols (TGs) [TG 48:2, TG(14:0/16:1/18:1); TG 48:3; TG 50:4, TG(16:1/16:1/18:2); TG 51:2; TG 51:4; TG 52:4, TG(16:0/18:2/18:2); TG 53:4; TG 56:6, TG(16:0/18:1/22:5); TG 58:6; TG 58:8; TG 60:9; TG 62:11]. Thirty-five lipids were lower in the CSF from probable iNPH patients (Fig. 2): three PEs (PE 38:4, PE 38:6 and PE 40:6), two plasmenyl-PEs (plasmenyl-PE 40:6 and plasmenyl-PE 43:6), one platelet-activating factor (docosahexaenoyl PAF C-16), three SMs (SM 32:3, SM 36:1 and SM 36:2), one ceramide (Ce 20:5), three from the small group collection, two PAs (PA 43:4 and PA 45:4), one plasmenylphosphatidylcholine (plasmenyl-PC) (plasmenyl-PC 40:6), one lysophosphatidylcholine (LysoPC 17:0), two PSs (PS 39:0 and PS 39:7) and 16 PCs [PC 30:0, PC 32:0,PC(16:0/16:0), PC 33:0, PC 34:1, PC 34:4, PC 36:1, PC 36:1.1, PC 36:2, PC 36:6, PC 38:1, PC 38:4, PC 38:6, PC 39:6.1, PC 40:4, PC 40:6, PC 40:7].

**Figure 3:**
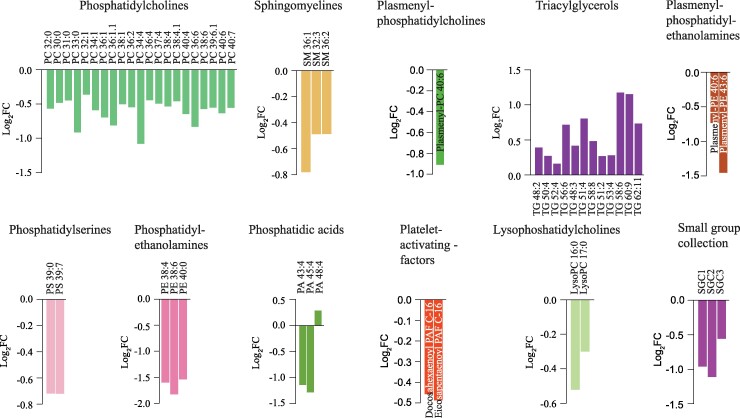
**Isolated lipid class analysis.** The difference (log_2_FC) lipid content within each lipid class in isolation between control subjects and patients with probable iNPH plotted as bars (log_2_ transformed), arranged according to their descending abundance. Data are based on lumbar CSF from 19 patients with probable iNPH and lumbar CSF from 21 control subjects. FC, fold change.

Determination of the lipid content within each lipid class in isolation unveiled significantly altered lipid abundance between CSF from probable iNPH patients versus control subjects within 11 of the main lipid classes (Fig. 3; Supplementary Table 2), where the majority of lipids displayed lower abundance in probable iNPH patients (e.g. LysoPCs, phosphatidylethanolamines, PSs, PAs, PCs, SMs, plasmenyl-PCs and plasmenyl-PEs). Only lipids from the class of TGs displayed elevation in CSF from patients with probable iNPH compared with control individuals. On the whole, several CSF lipids emerged with altered abundance in patients with probable iNPH, mainly evident as a lowering in abundance.

### No distinct CSF lipid profile in shunt-responsive probable iNPH patients

**Figure 4:**
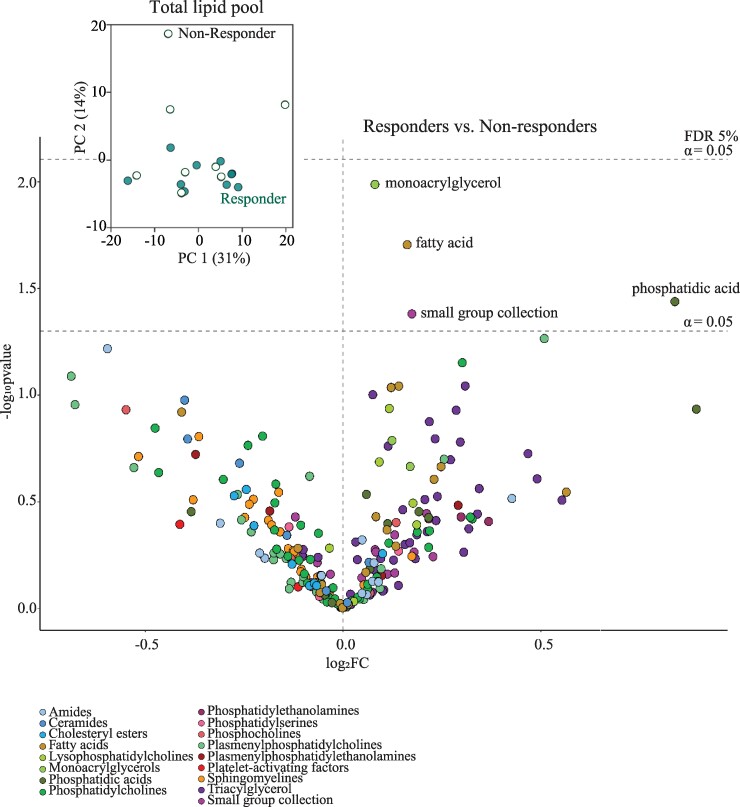
**CSF lipid distribution versus shunt responsiveness of probable iNPH patients.** Volcano plot of individual CSF lipids identified with the fold change (log_2_ transformed) between responders and non-responders (inset; PCA plot of total lipid values from shunt-responsive probable iNPH patients versus the non-responders). The upper dashed line indicates cut-off for significance (at a false discovery rate ≤ 0.05). Statistical evaluation was done with Welch’s *t*-test followed by the Benjamini–Hochberg method [with an adjusted *P* ≤ 0.05 (false discovery rate of 5%]. Data are based on lumbar CSF from 19 patients with probable iNPH.

All enrolled probable iNPH patients received a ventriculo-peritoneal shunt. Eleven of the 19 enrolled probable iNPH patients were categorized as ‘responders’ due to improvement in clinical symptoms following ventriculo-peritoneal shunt insertion, whereas the remaining eight probable iNPH patients were classified as ‘non-responders’ (see Materials and methods for details). To assess a potential correlation between CSF lipid content in patients with probable iNPH and their shunt responsiveness, we conducted a comparison between two groups of subjects: responders (those who positively responded to shunting) and non-responders (those who did not respond favourably to shunting). A PCA plot of the overall lipid content was employed to detect the potential separation of the two cohorts and illustrated an indistinguishable CSF lipid composition between responders and non-responders, as depicted in Fig. 4, inset, Supplementary Fig. 2 and Supplementary Table 3. To reveal whether the abundance of specific individual lipids was altered, but masked within the overall lipid pool, we employed a volcano plot analysis, which detected no statistically significant disturbance of lipid content in the CSF from shunt-responsive probable iNPH patients versus those that were non-responders (Fig. 4). However, four lipids (MG 20:2, PA 49:4, ethyl oleate and DG 36:4) appeared increased in non-responders before correction for multiple testing.

## Discussion

We here characterized the lumbar CSF lipid profiles in both control individuals and patients with probable iNPH, shedding new light on the CSF lipidome in physiological and in a neuropathological context with an unresolved aetiology. Our analysis detected a total of 358 compounds in CSF, with 244 of them belonging to 17 primary lipid classes. In control CSF, the lipid classes TGs, PCs, SMs, fatty acids and plasmenyl-PCs were found to display the highest number of different lipids within their respective class, whereas fatty acids, PCs and SMs were detected with the highest concentration. The CSF lipid distribution was independent of sex, both when analyzed collectively and when categorized into different lipid classes, as earlier demonstrated for cisternal CSF samples.^12^ The lipid group distribution presented with a biphasic age dependency for the majority of lipid classes with similar peaks around the mid-50s and the mid-70s. Two lipid classes were fairly stable across the various ages (fatty acids and monoacylglycerols), which aligns with the slight fluctuation in fatty acids and monoacylglycerols observed in cisternal CSF obtained from a cohort of patients undergoing vascular clipping of unruptured aneurysms.^12^ However, much higher sample sizes are required to fully delineate the age-dependent CSF lipid content. Previous research has demonstrated an age-dependent variation in the lumbar CSF lipid profile for plasmenyl-PEs, fatty acids and PCs in control subjects. These earlier samples were divided into age groups using a younger median split (40 years)^20^ in contrast to the present study (with a median split of 65 years).

We observed significant alterations in the CSF lipid levels in probable iNPH patients compared with those in control subjects, specifically within the main lipid class of TGs, of which 12 were increased in probable iNPH patients [TG 48:2, TG(14:0/16:1/18:1); TG 48:3; TG 50:4, TG(16:1/16:1/18:2); TG 51:2; TG 51:4; TG 52:4, TG(16:0/18:2/18:2); TG 53:4; TG 56:6, TG(16:0/18:1/22:5); TG 58:6; TG 58:8; TG 60:9; TG 62:11]. Triacylglycerols are known to rapidly cross the blood–brain barrier and have been associated with cognitive decline.^21^ Their increased abundance in CSF has also been observed in other neurological conditions, such as medulloblastoma-induced hydrocephalus^22^ and Alzheimer’s disease.^23^ However, the precise mechanisms underlying the alteration of CSF triacylglycerols levels in neurological disorders remain unclear and may involve increased brain cell activity or changes in blood–brain barrier function.^24,25^ Although the physiological role of these lipids in probable iNPH patients remains uncertain, it raises the possibility of using TGs as potential diagnostic markers to differentiate iNPH from other neurological diseases as for example Alzheimer’s disease: the two triacylglycerides associated with Alzheimer’s disease, TG 56:8 and TG 56:9,^23^ were unchanged in the CSF from probable iNPH patients compared with control subjects (this study). In probable iNPH patients, CSF lipids from other classes exhibited significant reduction. These reductions may have an impact on numerous peripheral signalling proteins associated with—or acting on—the plasma membrane, effectively rendering these lipids that are reduced in abundance as potential ‘lipid switches’. These activated responses could potentially initiate the formation of lipid droplets within the choroid plexus,^26^ the specialized tissue responsible for the majority of CSF production (for review, see MacAulaye *et al*.^27^).

Several PCs exhibited reduced abundance in the CSF of probable iNPH patients, including PC 30:0, PC 31:0, PC 32:0, PC 33:0, PC 32:1, PC 34:1, PC 36:1, PC 36:1.1, PC 38:1, PC 36:2, PC 34:4, PC 36:4, PC 37:4, PC 38:4, PC 38:4.1, PC 40:4, PC 36:6, PC 38:6, PC 39:6.1, PC 40:6 and PC 40:7. These lipids play crucial roles in signalling cascades, brain structure and function, potentially influencing the balance between cell survival and death.^28^ Reduced levels of PCs may be linked to aberrant phospholipase A2 activity, an enzyme responsible for cleaving fatty acids from the sn-2 position of phospholipids, resulting in the production of free fatty acids and LysoPCs.^29^ Consequently, we observed a reduction of two LysoPCs, LysoPC 16:0 and LysoPC 17:0 in probable iNPH patient CSF. These lipids are essential for maintaining cell membrane integrity, and alterations in their levels can lead to neuronal damage and cell loss, akin to observations in the CSF of Alzheimer's disease patients where the levels of LysoPCs tended to be lower than in CSF from controls, although no statistical difference was reached.^11^ Lysophosphatidic acid, derived from LysoPC, exhibited reduced CSF levels in patients with probable iNPH, which have been associated with neuropsychological functions and neuropsychiatric diseases.^30^ Additionally, the less abundant PAs were significantly reduced in probable iNPH patients. These are the simplest diacylglycerophospholipids and serve as anionic bioactive molecules that play crucial roles as second messengers in signalling pathways and as common intermediates in the synthesis of all phospholipids.

We identified a reduced abundance of three PAs (PA 43:4, 45:4 and 48:4). Plasmalogens, which are crucial for brain function,^31,32^ exhibited reduced levels, including plasmenyl-PEs 40:6 and 43:6, as well as plasmenyl-PC 40:6 in probable iNPH patients. Additionally, four SMs (SM 36:1, SM 32:2, SM 32:3 and SM 36:2) were of lower abundance in CSF from probable iNPH patients compared with that of control subjects. These sphingolipids play pivotal roles in membrane structure, fluidity, intercellular communication, signal transduction and cell activation.^33^ Altered sphingolipid metabolism is associated with neurodegenerative diseases like Alzheimer's disease.^13^ Furthermore, we observed reductions in three PSs (PS 39:0 and PS 39:7) and three PEs (PE 38:4, PE 38:6 and PE 40:0). PS, synthesized from serine and PEs, has been shown to improve cognitive measures in Alzheimer's disease,^34^ a clinical symptom also observed in iNPH patients. These findings suggest potential connections between reduced PSs and cognitive decline in iNPH.

### Limitations

In this study, we utilized a mass spectroscopy-based platform to comprehensively assess the complete lipid content in CSF from patients diagnosed with probable iNPH and control subjects. However, certain limitations need to be considered. We employed CSF samples from probable iNPH patients who were selected for shunting and did not include patients who did not receive a shunt. Besides the low sample number, we were, in addition, unable to reveal the exact time elapsed between the onset of iNPH symptoms and the collection of CSF samples, which could potentially introduce variability in the lipid profiles of the probable iNPH patients. We quantified the lipid content in the CSF for both groups at a single time point, which limits our ability to capture potential temporal variations that may well occur during disease progress. Due to statistical requirements of correction for multiple testing necessitated by the extensive quantification of CSF lipids in an untargeted manner, there is a possibility of encountering false negatives. Some lipids that might have predictive value for iNPH outcomes or shunt dependency, if individually tested using a benchtop assay, may not have been identified in our unbiased quantification. In addition, the cellular origin of the probable iNPH-specific CSF lipid distribution remains unresolved and, therefore, cannot, at present, be used to assign cellular pathology or reveal potential aetiology of iNPH. Lastly, the altered lipid composition in CSF from probable iNPH patients may be pathological or adaptive and may not, in itself, have pathophysiological significance. The latter, however, should not prevent the usage of these as a potential future biomarker.

### Clinical relevance

Biomarkers that accurately capture the altered CSF dynamics associated with iNPH hold significant potential for enhancing clinical assessment and decision-making processes. Most importantly, reliable biomarkers could distinctly confirm the diagnosis of iNPH and differentiate the condition from other neurological conditions and thus enhance the treatment precision. Future validation of the present findings in larger cohorts, as a function of time, and with the inclusion of patients with differential diagnosis, such as Alzheimer’s disease, vascular dementia, or Lewy body dementia, will be pivotal to verify the specificity of the probably iNPH lipid profile prior to inclusion of the lipid profile of iNPH patients as a potential biomarker. In addition, specific iNPH lipid biomarkers may provide a marker of injury that could accelerate our delineation of the underlying aetiology of the pathology. Although the CSF lipid profile in probable iNPH patients may indicate the disease process, it did not correlate with the patients’ shunt responsiveness. The lipid profile may therefore be involved in the underlying aetiology but does not accurately reflect the potential for treatment by shunt insertion. Consequently, the CSF lipid profile obtained during the diagnostic workup cannot, at present, serve as a predictive indicator of shunting outcomes. However, it would be of future interest to compare the lipid profile of probable iNPH patients not selected for shunt surgery versus those that were.

## Conclusion

In conclusion, our study demonstrates that the lipid profile of control subjects encompasses lipids from 21 different classes, with no discernible dependence on sex or age. Interestingly, we identified a characteristic CSF lipid profile in patients with probable iNPH when compared with control subjects. However, we did not observe a correlation between this lipid profile and a positive shunt outcome. This finding encourages further investigations to determine whether any or a combination, of the identified lipids, may serve as biomarkers tailored to iNPH. Consequently, novel lipid biomarkers may contribute to unravelling the aetiology of iNPH and provide potential avenues for improved diagnostic and prognostic strategies in the future.

## Supplementary Material

fcae388_Supplementary_Data

## Data Availability

Scripts for the data analysis can be found at https://github.com/Sorennorge/MacAulayLab-iNPH-Metabolomics.
