# Supplementary material for: The CSF lipid profile in patients with probable idiopathic normal pressure hydrocephalus differs from control but does not differ between shunt responders and non-responders
Source: Brain Commun. 2024 Nov 5;6(6):fcae388. doi: 10.1093/braincomms/fcae388 (PMC11562123; doi:10.1093/braincomms/fcae388)
Supplement: fcae388_Supplementary_Data [file fcae388_supplementary_data.zip › Supplementary_Figures 1 and 2.pdf]

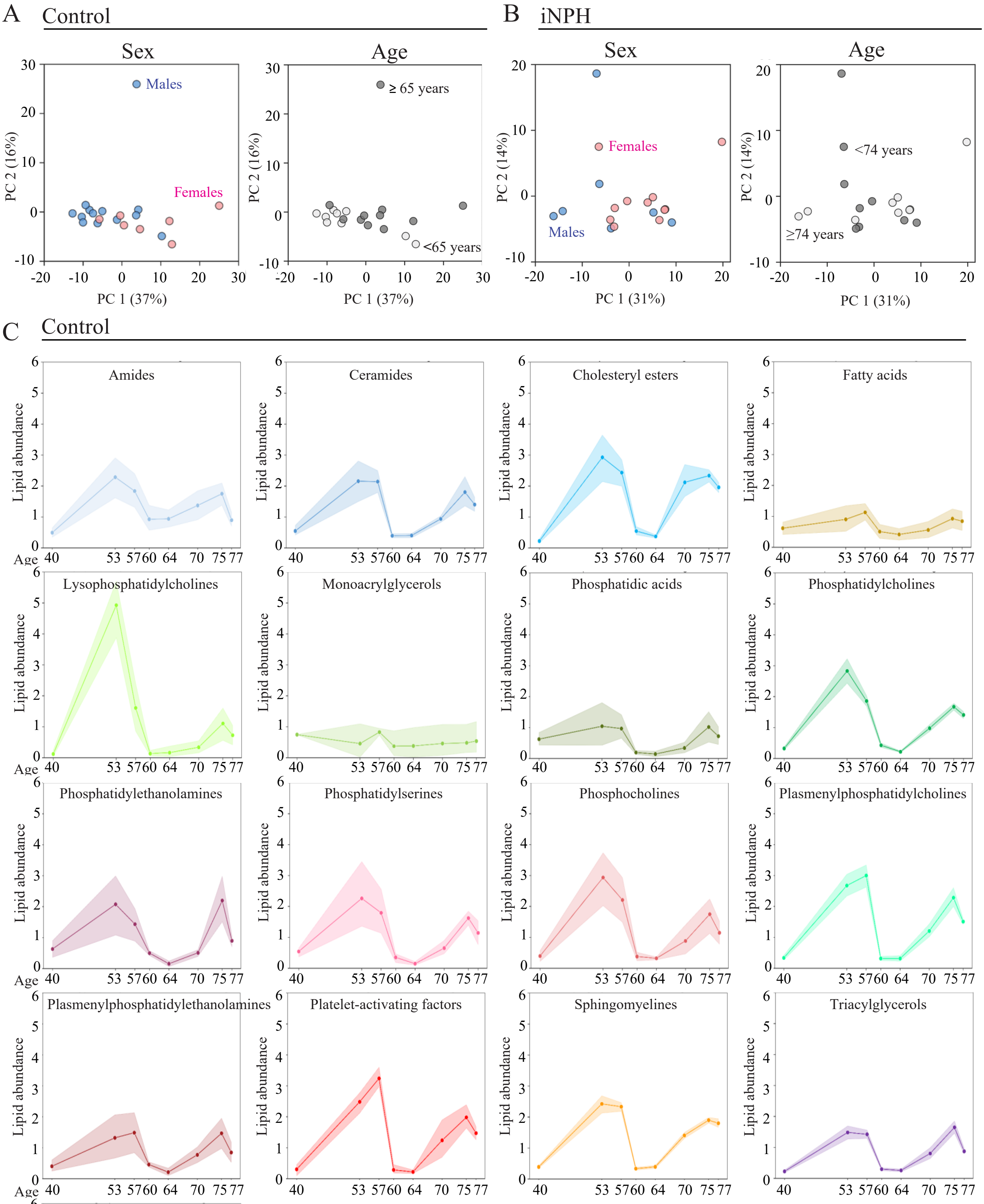

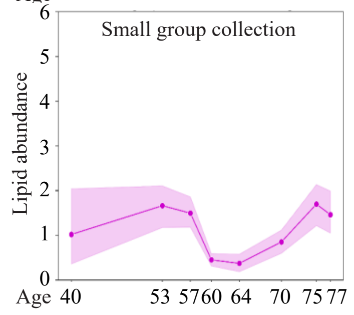

Supplementary Figure 1. Subject background A. The total amount of lipids grouped for sex plotted, and the total amount of lipids grouped for age initially divided at the median age) plotted in a PCA plot (controls). **B**. The total amount of lipids grouped for sex plotted, and the total amount of lipids grouped for age initially divided at the median age) plotted in a PCA plot (probable iNPH patients). **C**. Total lipid abundance fluctuation for each lipid group as a function of the age of the control subjects from the youngest (52 years old) to the oldest (84 years old). Data are based on lumbar CSF from probable iNPH patients (n=21) and lumbar CSF from control subjects (n=19).

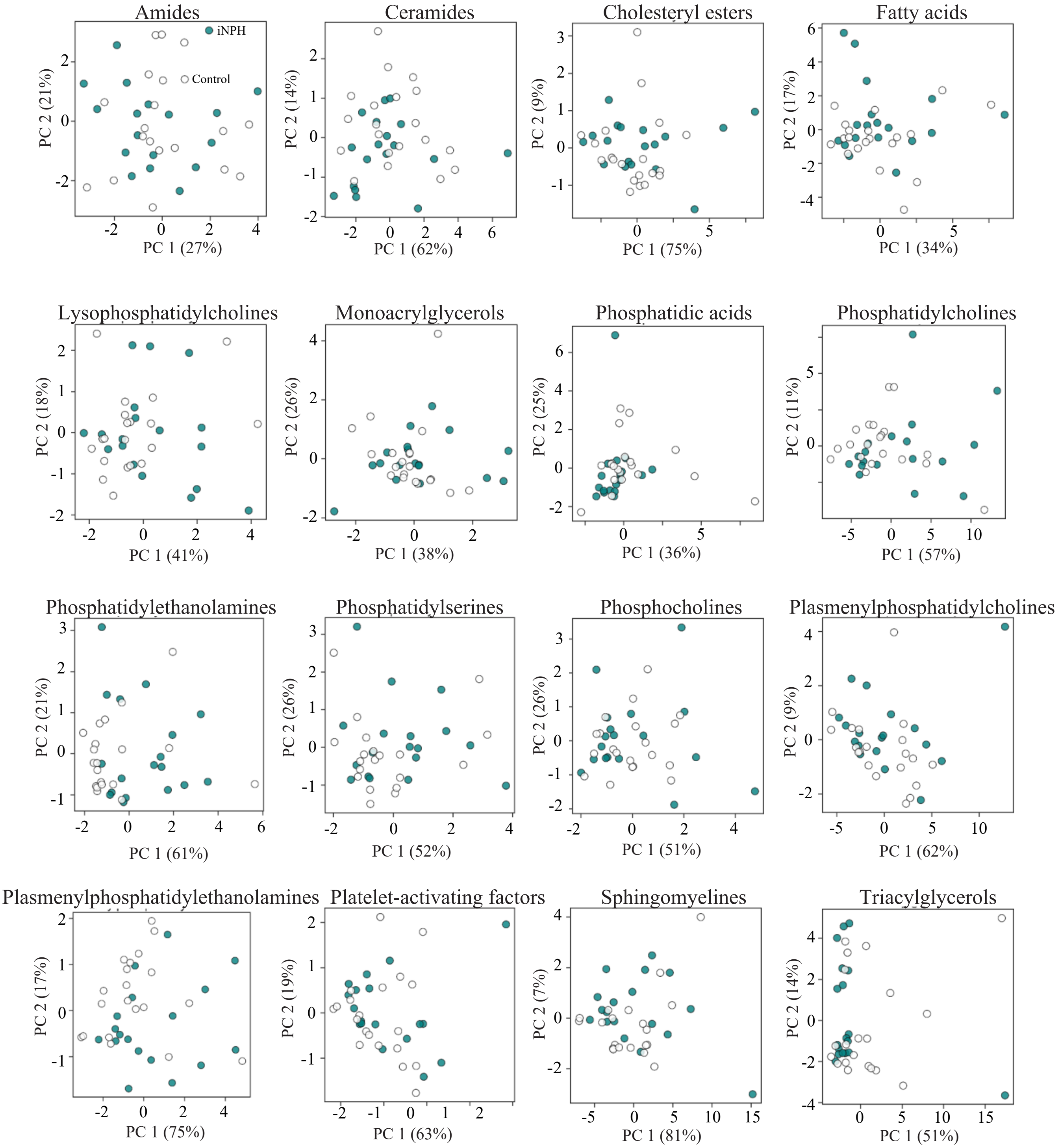

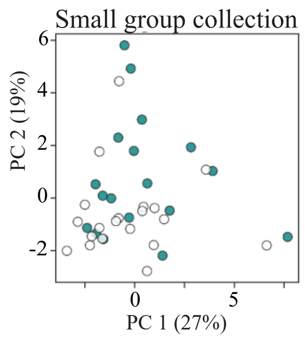

Supplementary Figure 2. CSF lipid levels in patients with probable iNPH compared to control subjects. The normalized individual lipid values were grouped for control subjects and patients with probable iNPH and plotted with respect to their first and second principal component (PC1 and PC2). Data are based on lumbar CSF from probable iNPH patients (n=21) and lumbar CSF from control subjcts (n=19).
